# Observations on the binding of testosterone to malignant mammary tumours and other tissues in vitro.

**DOI:** 10.1038/bjc.1967.83

**Published:** 1967-12

**Authors:** H. Braunsberg, V. H. James


### OBSERVATIONS ON THE BINDING OF TESTOSTERONE TO MALIGNANT MAMMARY TUMOURS AND OTHER TISSUES *IN VITRO*

HANNELORE BRAUNSBERG AND V. H. T. JAMES

*From the Department of Chemical Pathology, St. Mary's Hospital, London, W.2*

Received for publication July 25, 1967

THE marked clinical response of some patients with mammary carcinoma to endocrine ablation (Huggins and Bergenstal, 1951; Kennedy, 1956; Luft, Olivecrona, Ikkos, Nilsson and Ljunggren, 1956) or to androgen (McDonald, Gellhorn, Kennedy and Taylor, 1960; Segaloff, 1966) or oestrogen (McDonald, *et al.*, 1960) treatment leaves little doubt that the steroid hormones play an important part in tumour growth. Progesterone treatment alone does not appear to have any marked beneficial effect (Gordon, Horwitt, Segaloff, Murison and Schlosser, 1952; Volk, Escher, Huseby, Tyler and Cheda, 1960). At present, the mechanism of action of these substances is unknown. They may act directly at the tissue site, or they may exert an indirect effect, such as influencing the tissue through some mediator carried in the blood stream.

Of these possibilities, local action is more amenable to study. It implies free entry of hormones into the tumour, and this has been demonstrated in human subjects following injections of testosterone (Ellis, Parker, Bulbrook and Deshpande, 1965), steroid oestrogens (Davis, Wiener, Jacobson and Jensen, 1963; Demetriou, Crowley, Kushinsky, Donovan, Kotin and MacDonald, 1964), and progesterone (Deshpande and Belzer, 1966). The results of these single-injection experiments also suggested the existence of kinetic differences in steroid turnover in various tissues, which could arise from differences in tissue steroid concentrations, or in the rates at which these substances can enter and leave the tissues, or both (Braunsberg and James, 1967). The information currently available on tissue concentrations of steroid hormones is extremely limited because of the considerable technical difficulty of estimating quantitatively the small amounts which are present. Nevertheless, as a step towards a fuller understanding of the mode of action of these substances, knowledge of their distribution in the various body tissues would be of considerable value. In particular, the study of hormone dependent tumours might prove rewarding by indicating whether their dependence is related to the ability to provide, by concentration, a local environment which might be conducive or inhibiting to the neoplastic process.

Since direct chemical analysis is still impracticable, an alternative approach to the problem is offered by attempting to equilibrate the tissue under study with labelled steroid tracers. If such an equilibrium can be achieved, comparison of total radioactivity in various tissues will offer an indication of relative concentrations, and additionally the effect of possible antagonists to steroid uptake becomes amenable to study.

This procedure, an extension of the isotope dilution technique, is in principle applicable to both *in vivo* and *in vitro* studies. Both these approaches have been investigated in relation to the problem of human breast tumours. In this paper, the results obtained with an *in vitro* technique are presented; the *in vivo* studies are reported elsewhere (Braunsberg, Irvine and James, 1967).

### MATERIALS AND METHODS

*Tissue samples.*—Tissues removed at operation were immediately placed in ice-cold physiological saline, brought to the laboratory as quickly as possible, and sliced thinly by hand on a ceramic tile. Samples of not less than ten and not more than 400 mg. (usually 30–50 mg.) were weighed rapidly on a torsion balance and transferred to small vials containing the incubation medium (see below). All incubations were begun within 1 hour after removal of tissues from the patient, except where otherwise stated.

*Counting equipment.*—$^{14}C$ and $^{3}H$ were counted in a liquid scintillation coincidence counter (Nuclear Chicago, Model 725), giving efficiencies of 65 and 20 % under single label conditions and 40 and 10 % under dual-label conditions. $^{24}Na$ was counted in a well-type counter with a plastic scintillator (kindly made available by Professor J. C. Waterlow) or in the liquid scintillation counter using the method described by Braunsberg and Guyver (1964).

*Glassware.*—Pipettes were cleaned in chromic acid, washed with water, followed by an aqueous solution of " Pyroneg " (Diversey (U.K.) Ltd., London, W.1), and rinsed with glass-distilled water. It was found that even after prolonged soaking in 10 % sodium hydroxide and cleaning by the above procedure, the incubation vials remained contaminated with tritium. All incubations were, therefore, carried out in new plastic-stoppered glass vials 1 in. (2·54 cm.) diameter (Johnson and Jorgenson, London, S.E.7). New glass vials of 0·6 in. (1·5 cm.) diameter (Johnson and Jorgensen) were used for $^{24}Na$ counting and " Pronase " hydrolyses.

*Reagents.*—Testosterone-1,2-$^{3}H$ (specific activity 153 $\mu$Ci/$\mu$g.), kindly supplied by Dr. Marcel Gut, was purified by chromatography in a Bush A system (Bush, 1961) for 11 hours. Oestradiol-17$\beta$-6,7-$^{3}H$ (specific activity 134 $\mu$Ci/$\mu$g.) and progesterone-1,2-$^{3}H$ (specific activity 97 $\mu$Ci/$\mu$g.) were gifts from the Endocrinology Study Section, National Institutes of Health, Bethesda, Md., U.S.A. Testosterone-4-$^{14}C$ (101 $\mu$Ci/mg.) was from the Radiochemical Centre, Amersham, Bucks. These steroids were used without purification. Inactive testosterone from the M.R.C. collection was kindly made available by Professor W. Klyne.

" Pronase ", a protease from streptomyces griseus (Nomoto and Narahashi, 1959; Nomoto, Narahashi and Murakami, 1960) was prepared by the Kaken Chemical Company, Tokyo, Japan, and obtained from Kingsley and Keith, London, E.C.4. The buffer (pH 8·2) consisted of a mixture of 50 ml. of an aqueous solution containing 309 mg. boric acid (B.D.H., Analar) and 109·5 mg. calcium chloride hexahydrate (Hopkin and Williams, Analar) and 4 ml. 0·1 N sodium hydroxide (B.D.H., Analar). The enzyme solution containing 10 mg. " Pronase " in 10 ml. buffer could be kept in a refrigerator for several days.

Sodium chloride was reagent grade (May and Baker). Methylene chloride (general purpose reagent, Hopkin and Williams) was used without purification. Toluene (Hopkin and Williams, Analar) was used without further purification for preparing the liquid scintillator. The other scintillator chemicals were from Nuclear Enterprises, Sighthill, Edinburgh. The scintillator consisted of p-terphenyl (0·3 %) and POPOP (1,4-bis-2-(5-phenyl)-oxazoyl benzene, 0·004 %) in toluene.

*Incubations.*—The following steroid concentrations were used, except where otherwise stated: testosterone 0·1 μg./100 ml.; oestradiol 0·1 μg./100 ml.; progesterone 0·5 μg./100 ml. To prepare the incubation media, the appropriate amount of steroid, dissolved in ethanol, was measured into conical flasks and the solvent removed under a stream of nitrogen. Sodium chloride (0·9 % in water) containing $^{24}NaCl$ to give approximately 10,000 counts/minute/ml. was added and the mixture kept at 37° C. for 1 hour to aid dissolution of the steroid. 3 ml. of the solution was pipetted into each of the incubation vials which were kept at 37–40° C. while the tissues were sliced. Upon addition of the tissue samples the vials were closed with polythene stoppers through which wide injection needles had been inserted. Up to sixteen incubation vials were fixed on a circular platform by means of clips and the needles connected to a manifold through which oxygen could be passed into the incubation media. The whole platform was placed in a thermostatic water bath at 40° C. and agitated by gentle horizontal movement. Incubations were continued for exactly 1 hour.

*Analysis of samples.*—The tissue slices were transferred to smaller glass vials by means of fine forceps and the residual $^{24}Na$ present in the tissue and in 0·1 ml. of its medium was counted. Thus the volume of medium left in and on each tissue sample could be calculated.

*" Pronase " hydrolysis.*—Tissue samples were suspended in 1 ml. borate buffer, 0·1 ml. " Pronase " solution added, the vials stoppered and shaken in a thermostatic bath at 60° C. Further additions of 0·1 ml. " Pronase " solution were made every 2–4 hours (except overnight) until a fine suspension remained. This took from a few hours to two days depending on the nature of the tissue.

The samples were extracted with 15 ml. methylene chloride, and 12 ml. of the organic solvent phase pipetted into clean counting vials and taken to dryness under nitrogen.

It was important to sample and extract the saline media immediately after incubation. When allowed to stand overnight, for example, the saline solution lost tritium by adsorption on the glassware, and it was found that some, but not all the adsorbed tritium could be recovered with hot alcohol. All saline samples were analysed in duplicate and the mean tritium concentrations used to calculate uptake ratios (see below).

*Counting.*—All samples were dissolved in 10 ml. scintillator and counted twice or three times, a total of at least 10,000 counts being accumulated. Background count rates were taken over periods long enough to minimise the error of the net count rates (Jarrett, 1946). Quench corrections were made by means of internal standards consisting of labelled steroids dissolved in benzene.

*Calculations.*—Tritium concentrations were expressed as counts/minute/ml. medium (A) and counts/minute/g. tissue (B) and uptake ratios calculated as B/A.

### RESULTS

### *Methods for determining uptake ratios*

The performance of the analytical method used was compared with oxygen bomb combustion (Payne and Done, 1958). Testosterone-1,2-$^{3}H$ (0·027 μCi) was added to samples of human breast tissue (29–38 mg.) and recoveries calculated after analysis using " Pronase ". In four experiments the mean recovery was 99·9 % (range 96·4–102·5 %). In the absence of tissue, oxygen bomb combustion gave a mean recovery of 74·1 % (range 63·0–85·3 %) in five experiments in which 0·01 or 0·05 $\mu$Ci were added to filter paper, and in the presence of muscle tissue (77–123 mg.), recoveries of 0·01 or 0·02 $\mu$Ci ranged from 65·7 to 88·0 % with a mean of 74·5 %. Addition of the labelled hormone to inactive tissue may not reflect the performance of these methods for tissue incubated with steroids in which the tritium may be distributed on and within the cells. The two methods were therefore compared for eight pairs of muscle samples incubated with testosterone-1,2-$^3$H. Using the " Pronase " method, the mean uptake ratio was 6·2 (range 4·9–8·7), whilst bomb combustion gave a mean of 5·8 (range 3·3–8·9). Thus the two methods gave similar recoveries and results for tissues incubated with labelled steroid.

The precision of the extraction method was further tested by analysing the results of 60 duplicate determinations of tritium concentrations in the medium following incubation with tissue. The mean concentration for these samples was 6760 counts/minute/ml. (range 3991–9551) and the within sample deviation calculated from differences between duplicates (Youden, 1951) was 240·6 counts/minute/ml. Thus the coefficient of variation was 3·6 %.

It was not possible to estimate the within sample errors of determining tritium in the tissues, since duplicate analyses were not possible. The variability of tissue concentrations is a combination of analytical errors and biological variation and the former will be at least as great as those found for the saline samples.

*Conditions for collecting and incubating tissues*

Since the tissues could not be prepared for incubation immediately after removal from the patients, it was important to know what effect, if any, the delay may have on tissue uptake ratio. Tissue samples were sliced and incubated immediately upon arrival in the laboratory and after additional periods up to 3 hours in ice-cold saline. The results indicated that uptake ratios may change, or become more variable, when tissues are kept for longer periods. Samples for which the time between collection and incubation exceeded 1 hour have, therefore, been excluded, but it was rarely possible to start incubation in less than half an hour after removal of tissues from the patient.

Two experiments were carried out to examine the need of oxygenation during incubation with labelled testosterone. In one case, in which incubation was begun 30 minutes after removal of tissues from the patient, the uptake ratios, determined in triplicate, were 11·6, 11·2 and 10·0 (mean 10·9) with oxygen bubbled through the medium, and 7·2, 12·3 and 3·2 (mean 7·6) in the absence of oxygen. In the second experiment, in which incubations were started ninety minutes after collection, the uptake ratios were 8·5, 10·7 and 8·2 (mean 9·1) with oxygen bubbled through the medium, and 4·3, 4·5 and 5·1 (mean 4·6) with oxygen blown over the surface of the medium. It would seem, from these limited observations, that inadequate supply of oxygen to the tissues may reduce uptake ratios or make them more variable.

The effect of deep-freezing tissues was studied in two experiments with labelled testosterone. Uptake ratios were 2·8 and 3·5 for a sample of fresh breast cancer tissue. After 8 and 15 days at −20° C., mean uptake ratios of 4·8 (range 3·9 to 5·5) and 5·1 (range 4·3 to 5·9), respectively, were found. These differences were just significant ($p < 0·05$ and $p < 0·1$, respectively). For a sample of hypertrophied prostate, mean uptake ratios were 7·3 (range 6·4–8·4) for fresh tissue and 6·5 (range 5·6—7·3) after 14 days at —20° C. The difference was not significant. Because of the possibility of changes in steroid uptake following deep-freezing only fresh tissue samples were investigated.

To test whether dead tissue could still take up steroids from a saline medium, samples of breast cancer tissue were incubated after 1·6–2 hours in ice cold saline and after 4·5 hours at 40° C. In one experiment, testosterone uptake ratios were 8·3 and 7·1 for fresh tissue and 4·9, 4·1 and 4·0 after pre-incubation, the difference being significant ($p < 0{\cdot}005$). In a second experiment, progesterone uptake ratios were 15·0 and 27·3 for fresh tissue and 12·4 and 16·6 after pre-incubation; the difference was not significant. It is possible that greater differences might have arisen by comparison with tissues kept in ice-cold saline for shorter periods.

The effect of steroid concentration in the incubating medium on uptake ratios was examined for testosterone, progesterone and oestradiol. The results, which are shown in Table I, indicate that no significant differences could be demonstrated for the tissues and concentrations tested.

TABLE I.—*Effect of Steroid Concentration on Uptake Ratios*

Tissue slices were incubated for 1 hour at 37° C. with different concentrations of steroids in 0·9% NaCl

| Tissue | Steroid | Concentration (μg./100 ml.) | Uptake ratios | Significance |
|---|---|---|---|---|
| Muscle | Testosterone | 0·05 | 1·9, 1·1 | $p < 0{\cdot}1$ |
| | | 0·1 | 2·2, 2·5 | |
| | | 0·5 | 4·1, 2·8 | N.S. |
| | Progesterone | 0·5 | 13·8, 12·7 | N.S. |
| | | 1·0 | 34·6, 17·3 | |
| | Oestradiol | 0·05 | 12·2, 7·6 | N.S. |
| | | 0·1 | 8·7, 14·9 | |
| | | 0·5 | 16·5, 14·4 | N.S. |
| Carcinoma of breast | Testosterone | 0·1 | 2·8, 3·5 | N.S. |
| | | 0·5 | 1·3, 3·3 | |
| | Progesterone | 0·5 | 17·7, 14·0 | N.S. |
| | | 1·0 | 16·9, 10·2 | |
| | Oestradiol | 0·1 | 6·7, | — |
| | | 0·5 | 8·2, 11·8 | |
| Adipose | Testosterone | 0·1 | 52·9, | — |
| | | 0·5 | 59·3, | |
| | Progesterone | 0·5 | 147·4, 217·3 | N.S. |
| | | 1·0 | 178·2, 175·0 | |
| | Oestradiol | 0·1 | 76·9, 104·6 | N.S. |
| | | 0·5 | 119·7, 88·1 | |

*Tissue steroid uptake ratios*

The testosterone uptake ratios of twenty-three tissue samples from sixteen patients are shown in Table II and summarised in Fig. 1. Of the normal tissues examined, adipose tissue took up the largest and muscle and breast tissue the smallest amounts of steroid. The prostate tissue of two elderly men did not concentrate testosterone to any marked extent. Three of the nine samples of breast cancer tissue studied took up significantly more testosterone than muscle, but since many of these tumours contain considerable proportions of adipose tissue, these results do not clearly indicate an affinity for testosterone of the tumour cells. The remaining six tumours gave uptake ratios similar to those for normal breast tissue. The sample from patient 4 consisted of fibrotic breast tissue only, with no evidence of malignant cells.

FIG. 1. Testosterone uptake ratios of tumour samples from patients 1 to 10 and of normal tissues. A = adipose tissue; M = muscle; NB = normal breast tissue; P = prostate. Each column represents a mean, and vertical lines show the ranges of values.

It is possible that these uptake ratios do not reflect concentration of testosterone, but of a metabolic product formed during the incubation. To test this, samples of tumour tissue were incubated in the usual way and re-incubated in saline containing inactive testosterone (0·1 $\mu$g./100 ml.) for periods of 3, 10, 30 and 60 minutes. After 60 minutes, only about 15 % of the original radioactivity was associated with the tissue while at 3 minutes over 70 % was still present in the tissue. A known amount of testosterone-4-$^{14}$C was added to each of the saline

TABLE II.—*Tissue Testosterone Uptake Ratios*

| Case No. | Sex | Age | Diagnosis | Tissue | Uptake ratios |
|---|---|---|---|---|---|
| 1 | ♂ | 69 | Carcinoma of breast | Tumour | 4·7, 4·5, 5·7 |
| 2 | ♀ | 59 | Carcinoma of breast | Adipose<br>Tumour | 40·9, 47·0, 51·8<br>9·5, 7·6, 6·9 |
| 3 | ♀ | 68 | Carcinoma breast | Normal breast<br>Tumour | 7·4, 5·3, 7·7*<br>35·5, 21·8, 27·5* |
| 4 | ♀ | 41 | Carcinoma† of breast | Breast (fibrosis)<br>Muscle | 2·8, 2·0, 6·2<br>4·6, 5·7, 4·5 |
| 5 | ♀ | 75 | Carcinoma of breast | Normal breast‡<br>Tumour | 7·4, 4·0<br>6·4, 8·4, 6·6 |
| 6 | ♀ | 58 | Carcinoma of breast | Normal breast¶<br>Tumour | 9·0, 6·4, 6·3<br>3·9, 6·4, 6·5 |
| 7 | ♀ | 63 | Carcinoma of breast | Adipose<br>Tumour | 42·9, 42·6<br>10·1, 10·4 |
| 8 | ♀ | 63 | Carcinoma of breast | Tumour | 2·8, 3·5 |
| 9 | ♀ | 65 | Carcinoma of breast | Tumour | 10·4, 11·6, 9·6 |
| 10 | ♀ | 32 | Carcinoma of breast | Tumour | 6·1, 5·7, 6·3 |
| 11 | ♂ | 75 | Prostatectomy | Prostate | 11·6, 11·2, 10·0 |
| 12 | ♂ | 77 | Prostatectomy | Prostate | 8·4, 7·1, 6·4 |
| 13 | ♂ | 36 | Amputation | Muscle | 7·1, 7·4, 6·8 |
| 14 | ♀ | 30 | Regional perfusion | Muscle | 8·0, 6·7 |
| 15 | ♂ | 66 | Aneurism | Adipose | 52·7, 40·0 |
| 16 | ♀ | 61 | Papilloma of rectum | Rectal muscle<br>Benign polypus | 3·8, 4·7, 4·4<br>4·8, 4·9 |

* Incubation medium contained 0·4 μg./100 ml. testosterone.
† This patient had had her tumour removed previously and had been given X-ray therapy.
‡ Contained duct tissue with pink cell metaplasia.
¶ Collagen with an occasional small atrophic structure that is probably a duct.

samples, which were then extracted with methylene chloride. The extracts were evaporated to dryness and the residues partitioned between petrol and 80% methanol. Residues from the methanol layer were chromatographed on paper in a Bush A system for 6 hours. The eluted testosterone regions were counted for $^{14}C$ and $^{3}H$ and the $^{3}H$ count rate corrected for losses of $^{14}C$. Of the crude total tritium present in the salines 115·7, 107·8, 98·0 and 94·5% was recovered as testosterone for the 3, 10, 30 and 60 minute incubations respectively. There was, therefore, no evidence for metabolism of testosterone by the small tumour samples used.

In this experiment it could also be shown that exchange of testosterone with the tissue was slower than that of Na ion. Thus the $^{24}Na$ taken up by the slices left the tissue much more rapidly than did the steroid, the proportion remaining at 3 minutes being about 6% and at 60 minutes less than 1%.

The uptake of three steroid hormones has been compared for samples of muscle, breast tumour and prostatic tissues and the results are shown in Table III. Uptake ratios were highest for progesterone for all three samples. With the exception of prostatic tissue, uptake of oestradiol was higher than that of testosterone.

TABLE III.—*Uptake Ratios for Different Steroids*

Mean ratios are shown in brackets

| Case Number | Tissue | Uptake ratios: Oestradiol | Progesterone | Testosterone |
|---|---|---|---|---|
| 8 | Tumour | 8·2,* 11·8* (10·0) | 17·7, 14·0 (15·9) | 2·8, 3·5 (3·2) |
| 12 | Prostate | 6·6, 8·2, 13·8 (9·5) | 49·3, 128·4, 51·8 (76·5) | 8·4, 7·1, 6·4 (7·3) |
| 14 | Muscle | 14·7, 15·9 (15·3) | 21·5, 17·6 (19·6) | 8·0, 6·7 (7·4) |

* 0·5 $\mu$g./100 ml.

To test whether oestradiol could affect uptake of testosterone, samples of breast tumour tissue were incubated with labelled testosterone in the presence or absence of oestradiol (0·42 and 4·1 $\mu$g./100 ml.). In neither case could a significant difference be demonstrated. Testosterone uptake by muscle and prostate was not influenced by oestradiol (4·1 $\mu$g./100 ml.). In a further experiment, in which prostatic tissue was incubated with labelled oestradiol in the presence and absence of testosterone (0·5 $\mu$g./100 ml.), no significant difference could be demonstrated.

To study the effect of protein on the *in vitro* uptake of steroids by tissues, samples of tumour, muscle and adipose tissue were incubated in the presence or absence of albumin (4 %). The results are shown in Table IV. A sample of adipose tissue incubated with testosterone in the patient's plasma gave a ratio of 2·2.

TABLE IV.—*Effect of Protein on Steroid Uptake by Tissues*

| Tissue | Steroid | Uptake ratios: Without albumin | With albumin |
|---|---|---|---|
| Breast tumour | Testosterone | 18·7, 11·8 | 0·55, 1·85 |
| Breast tumour | Testosterone | 10·7 | 0·7, 0 |
| Muscle | Testosterone | 10·7, 17·8 | 2·3, 2·1 |
| Adipose tissue | Testosterone | 107·2, 97·8 | 6·7, 13·7 |
| Breast tumour | Oestradiol | 29·0, 30·3 | 0·6, 0·8 |
| Muscle | Oestradiol | 15·0, 16·6 | 0·5, 0·9 |
| Adipose tissue | Oestradiol | 207·0, 204·5 | 8·0, 8·0 |

### DISCUSSION

The validity of *in vitro* studies using the isotopic technique necessarily involves several major assumptions:

(1) Tissue slices behave in the same way as the whole tissue *in vivo* and all parts of the slice are completely accessible to the medium.
(2) The tissue preparations can be kept viable long enough to achieve complete equilibration.
(3) Concentrations of the labelled compound are similar to those normally surrounding the tissue *in vivo* or, if greater, do not alter the distribution of the compound in the tissue relative to the medium.

Quantitative comparisons between *in vitro* equilibration and single injection of the labelled substance *in vivo* (Stone and Baggett, 1965, 1966) are complex and would require careful mathematical analysis (Braunsberg and James, 1967). Analysis of tissue radioactivity after constant infusions of the labelled material *in vivo* provides a more convenient basis of comparison with *in vitro* equilibration experiments (Braunsberg and James, 1967). Such a study with labelled testosterone (Braunsberg, Irvine and James, 1967), like the *in vitro* studies reported here, failed to reveal any marked specific uptake of radioactivity by breast tumour tissue. The *in vitro* uptake of adipose tissue in relation to that of muscle appears to be rather higher than that *in vivo*.

The experiments in which tissues were re-incubated with inactive testosterone in saline, after uptake of the labelled material, show that exchange with the medium is virtually complete after one hour.

In their experiments with oestrogens, Stone and Baggett (1965) found that lack of oxygen had no effect on the amount of labelled steroid taken up during incubation, and concluded that the association between hormone and tissue was not energy dependent. The present experiments indicate that inadequate oxygenation of the tissues during incubation may lead to changes or greater variability in uptake ratios. This finding may merely reflect the need to keep the tissue viable during incubation, and does not necessarily implicate an energy-dependent process.

The hormone concentrations used in the experiments reported here were somewhat greater than those in human plasma. They may be far greater than those present in extracellular fluid. The results of the experiments in which albumin was added to the incubation medium indicate that much less unbound hormone may be available for entry into the tissues when plasma protein binding sites compete. When *in vitro* experiments are used to compare plasma and tissue binding, media containing plasma proteins may better simulate *in vivo* conditions. Nevertheless, experiments with protein-free media may indicate differences in hormone binding between individual tissues and between different steroids. That increased hormone concentrations did not materially affect the present results suggests that the number of available binding sites was not a limiting factor at the concentrations used here.

There is no evidence at present that the tissues examined are able to metabolise testosterone to any appreciable extent. No metabolic conversion of testosterone by human tumour could be demonstrated. King, Panattoni, Gordon and Baker (1965) found that hormone dependent and independent chemically induced rat breast tumours could metabolise testosterone. However, the substrate concentrations used by these workers were 10,000 times as great as those used in the present work and the yield of metabolite less than 0·5 %. It is unlikely, therefore, that the uptake ratios determined require correction for the presence of metabolic products.

That individual tumours may differ to a small extent in testosterone concentrations cannot be excluded. Thus, the precision of the method used was such that errors of up to 40–50 % in individual uptake ratios could occur. Such errors, together with variability of individual tissue samples, made it impossible to detect small differences. Any small effect of oestradiol on the testosterone uptake ratios of the tissues studied may also have escaped notice.

Tissue uptake ratios were highest for progesterone, lowest for testosterone, with adipose tissue giving the highest ratios of the tissues studied. It is possible that the affinity for steroids of adipose tissue is due to the lipids present, whereas that of the other tissues may be due to a protein. The differences in uptake ratios for different steroids suggest some specificity. The distribution of the steroid on or in the cells may also differ from one tissue to another and not all the steroid present may be physiologically active (cf. Sharp and Leaf, 1966). Thus apart from any doubt regarding the validity of these *in vitro* experiments, the physiological interpretation of their results is difficult and requires more detailed studies.

The relatively rapid entry of hormone into the tissue and the loss of a large proportion of the incorporated radioactivity during a short reincubation with inactive hormone indicates that a rapidly exchanging pool may exist in the tissues. Single injection or short-term infusion studies *in vivo* will similarly reveal only the rapid turnover of hormones in tissues. There may, however, be considerabe concentrations of bound hormones which, though not detected by these techniques, may be of great biological importance. It is as well to keep these limitations in mind, when using tracer methods to study turnover and concentrations.

Only one of nine malignant tumours could be shown to take up considerably more radioactivity than muscle and normal breast tissue. This might have been due to a high proportion of adipose tissue within the tumour substance. It is concluded that no marked binding of testosterone could be demonstrated in the majority of tumour samples under the conditions used in these *in vitro* studies.

### SUMMARY

The uptake of labelled testosterone, oestradiol and progesterone by malignant mammary tumours and normal tissues has been studied *in vitro*. Adipose tissue took up considerably more testosterone than muscle, normal breast, carcinoma and prostatic tissues. The highest uptakes were observed for progesterone. With the exception of prostatic tissue, uptake of oestradiol was higher than that of testosterone. There was no evidence that testosterone could influence the uptake of oestradiol or that the latter could affect the uptake of testosterone. Steroid uptake by tissue slices was considerably reduced or abolished by plasma proteins.

This work was supported by the British Empire Cancer Campaign for Research. The liquid scintillation spectrometer was made available by the Wellcome Trust and an oxygen combustion bomb was kindly lent by Dr. P. R. Payne. We wish to thank Professor W. T. Irvine for the opportunity to study his patients and Professor A. Neuberger for his kind interest in this work. Technical assistance was provided by Mr. A. Guyver.

### REFERENCES

BRAUNSBERG, H. AND GUYVER, A.—(1964) *Analyt. Biochem.*, **10**, 86.
BRAUNSBERG, H., IRVINE, W. T. AND JAMES, V. H. T.—(1967) *Br. J. Cancer*, **21**, 714.
BRAUNSBERG, H. AND JAMES, V. H. T.—(1967) *J. clin. Endocr. Metab.*, **27**, 1174.
BUSH, I. E.—(1961) 'The Chromatography of Steroids', Oxford (Pergamon Press).
DAVIS, M. E., WIENER, M., JACOBSON, H. I. AND JENSEN, E. V.—(1963) *Am. J. Obstet. Gynec.*, **87**, 979.
DEMETRIOU, J. A., CROWLEY, L. G., KUSHINSKY, S., DONOVAN, A. J., KOTIN, P. AND MACDONALD, I.—(1964) *Cancer Res.*, **24**, 926.

DESHPANDE, N. AND BELZER, F. O.—(1966) *Excerpta Medica*, Int. Congr. Series, **111**, 71.
ELLIS, F., PARKER, J. R., BULBROOK, R. D. AND DESHPANDE, N.—(1965) *Br. J. Surg.*, **52**, 54.
GORDON, D., HORWITT, B. N., SEGALOFF, A., MURISON, P. J. AND SCHLOSSER, J. V.—(1952) *Cancer, N.Y.*, **5**, 275.
HUGGINS, C. AND BERGENSTAL, D. M.—(1951) *J. Am. med. Ass.*, **147**, 101.
JARRETT, A. A.—(1946) *Rep. Congr. atom. Energy Commn U.S.*, A.E.C.U. 262.
KENNEDY, B. J.—(1956) *Am. J. Med.*, **21**, 721.
KING, R. J. B., PANATTONI, M., GORDON, J. AND BAKER, R.—(1965) *J. Endocr.*, **33**, 127.
LUFT, R., OLIVECRONA, H., IKKOS, D., NILSSON, L.-B. AND LJUNGGREN, H.—(1956) *Am. J. Med.*, **21**, 728.
McDONALD, I., GELLHORN, A., KENNEDY, B. J. AND TAYLOR, S. G.—(1960) *J. Am. med. Ass.*, **172**, 1271.
NOMOTO, M. AND NARAHASHI, Y.—(1959) *J. Biochem., Tokyo*, **46**, 653, 1481, 1645.
NOMOTO, M., NARAHASHI, Y. AND MURAKAMI, M.—(1960) *J. Biochem., Tokyo*, **48**, 453, 593.
PAYNE, P. R. AND DONE, J.—(1958) *Physics Med. Biol.*, **3**, 16.
SEGALOFF, A.—(1966) *Recent Prog. Horm. Res.*, **22**, 351.
SHARP, G. W. G. AND LEAF, A.—(1966) *Recent Prog. Horm. Res.*, **22**, 431.
STONE, G. M. AND BAGGETT, B.—(1965) *Steroids*, **5**, 809.—(1966) *Steroids*, **6**, 277.
VOLK, H., ESCHER, G. C., HUSEBY, R. A., TYLER, F. AND CHEDA, J.—(1960) *Cancer, N.Y.*, **13**, 757.
YOUDEN, W. J.—(1951) ‘ Statistical Methods for Chemists ’, New York (John Wiley and Sons), p. 16.


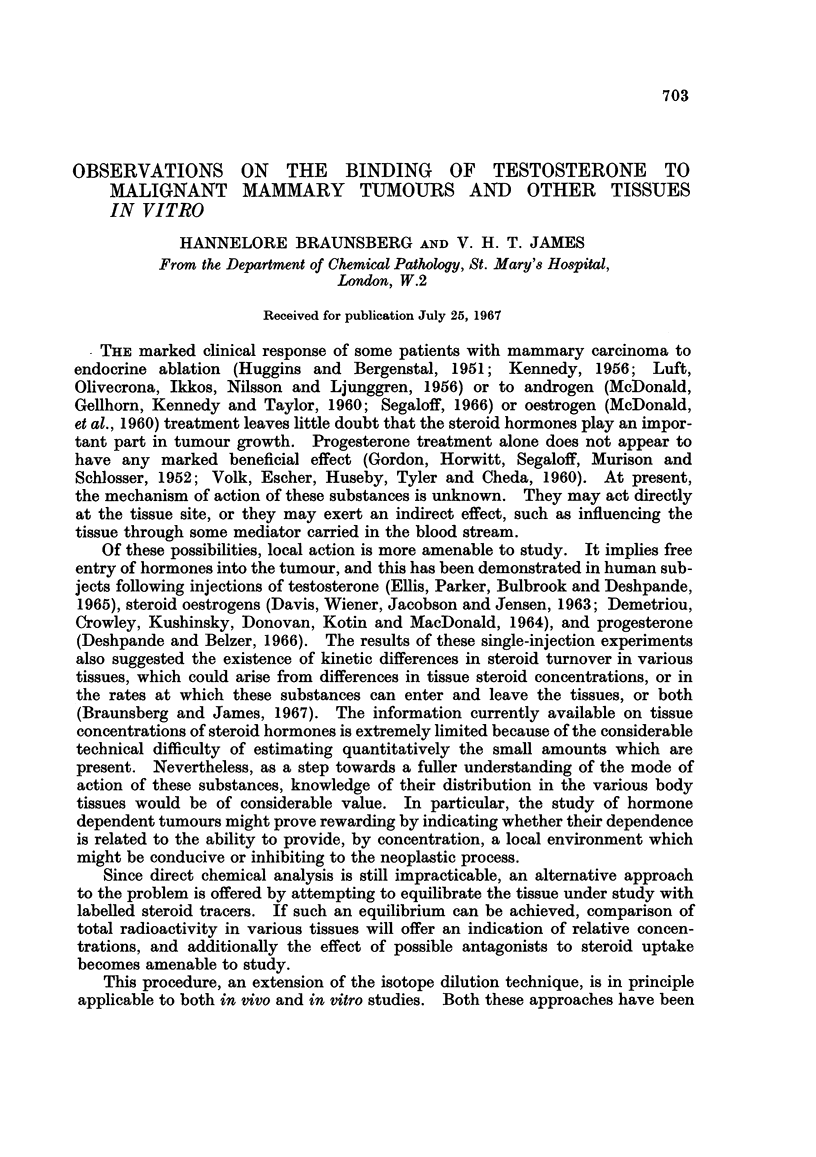

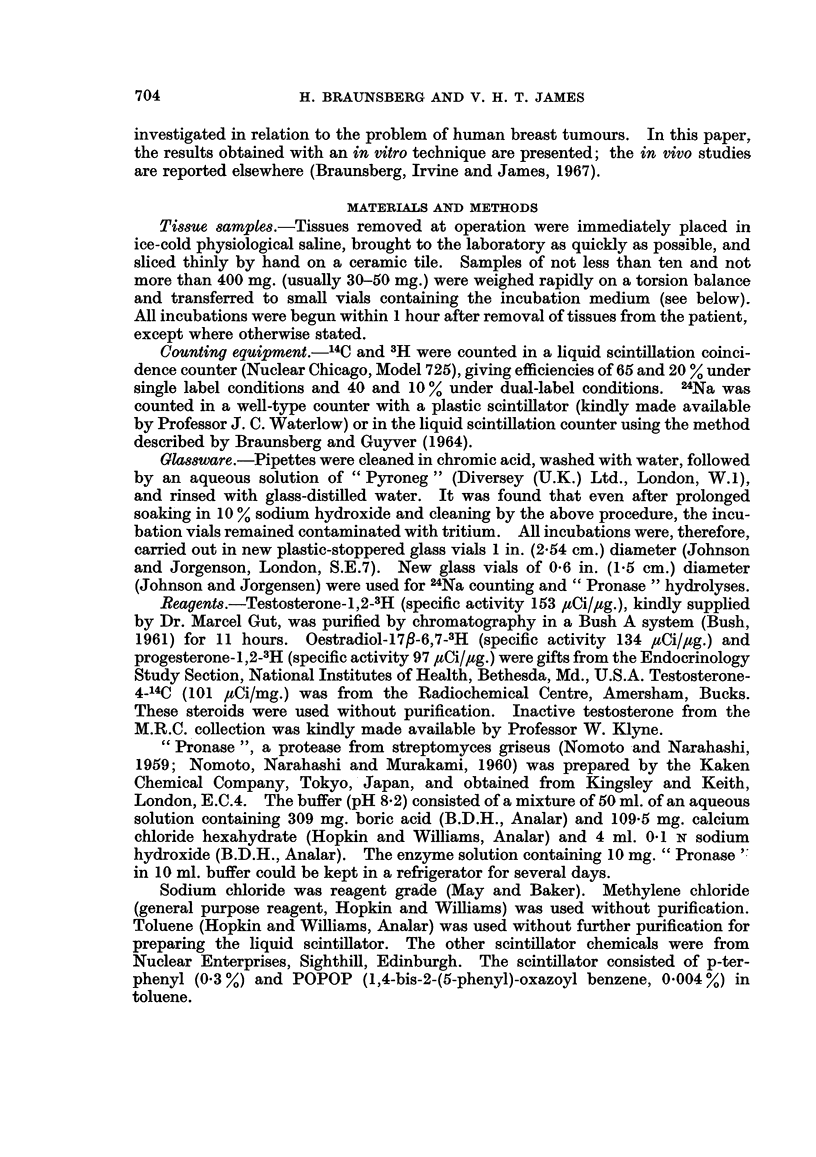

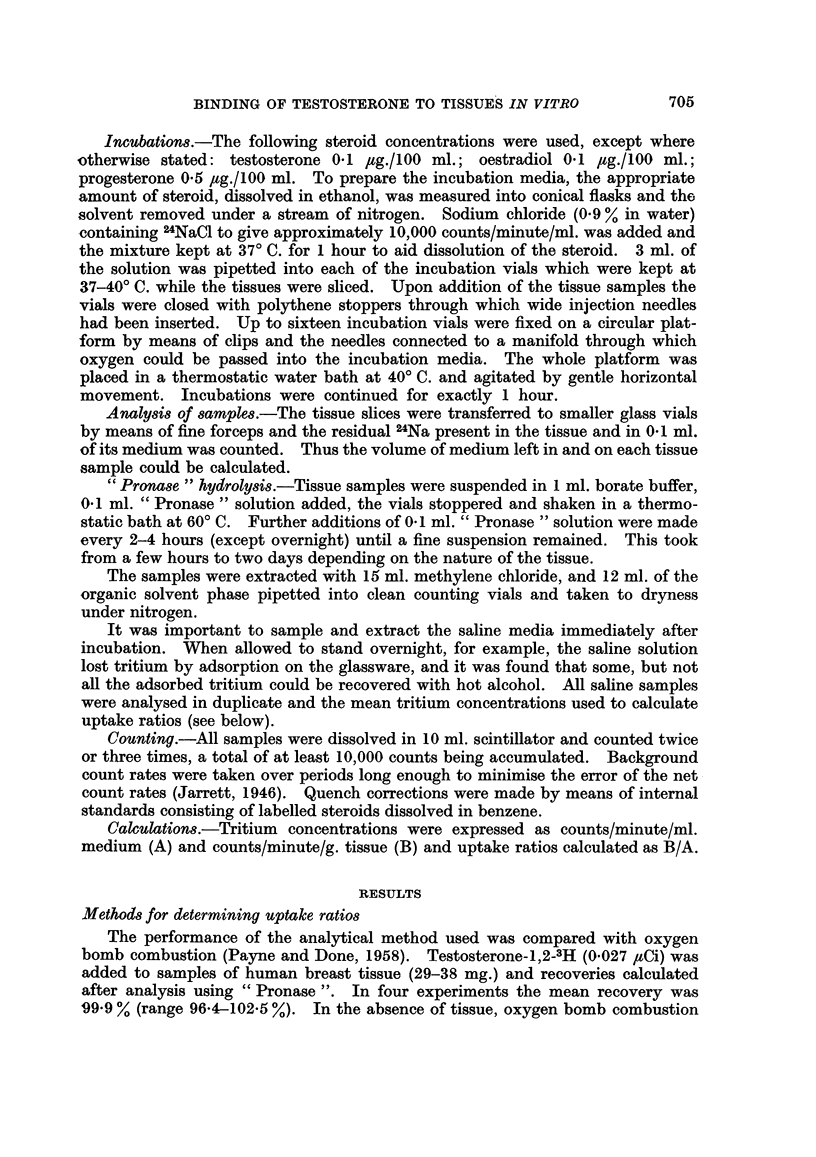

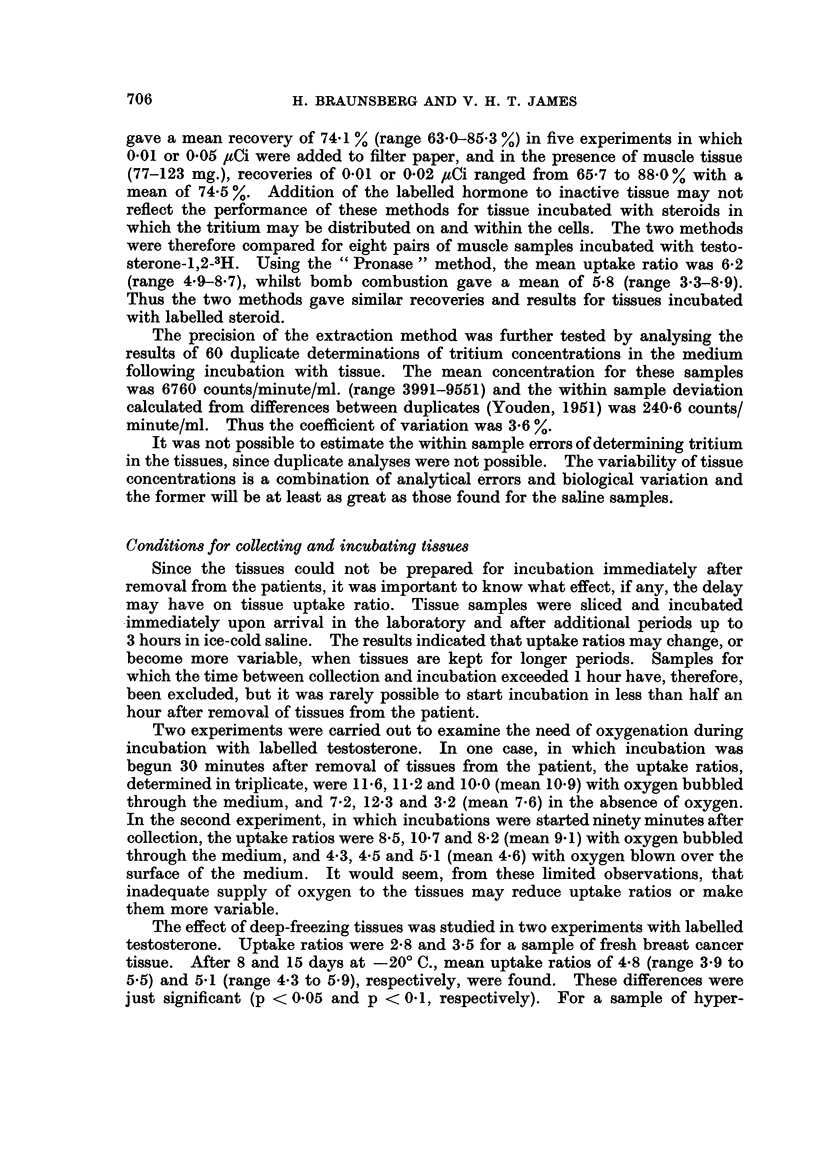

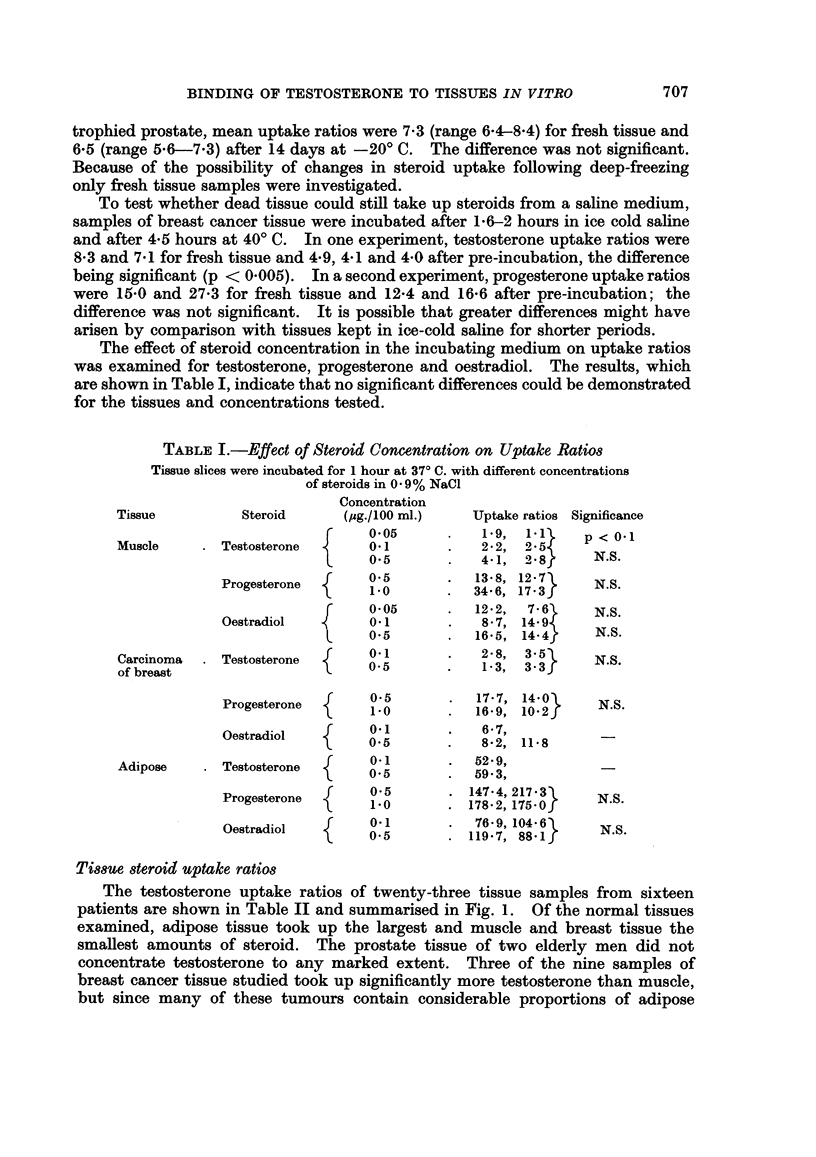

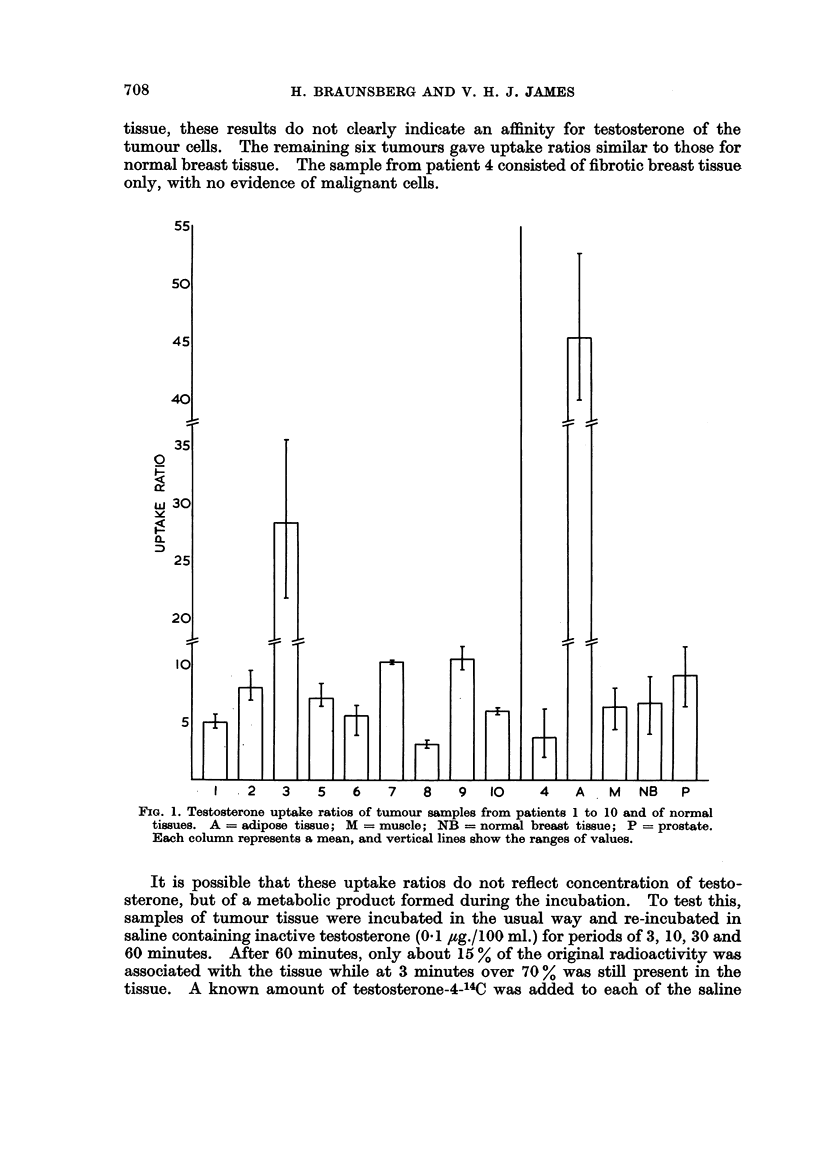

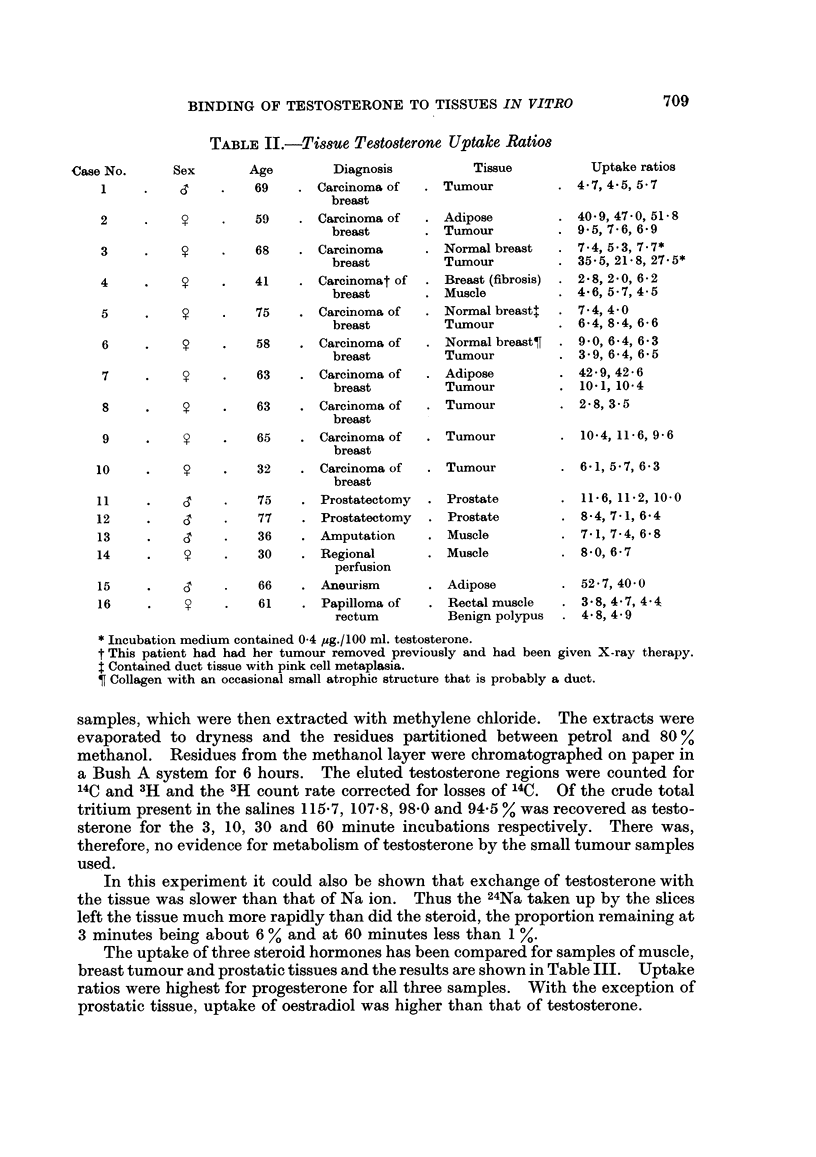

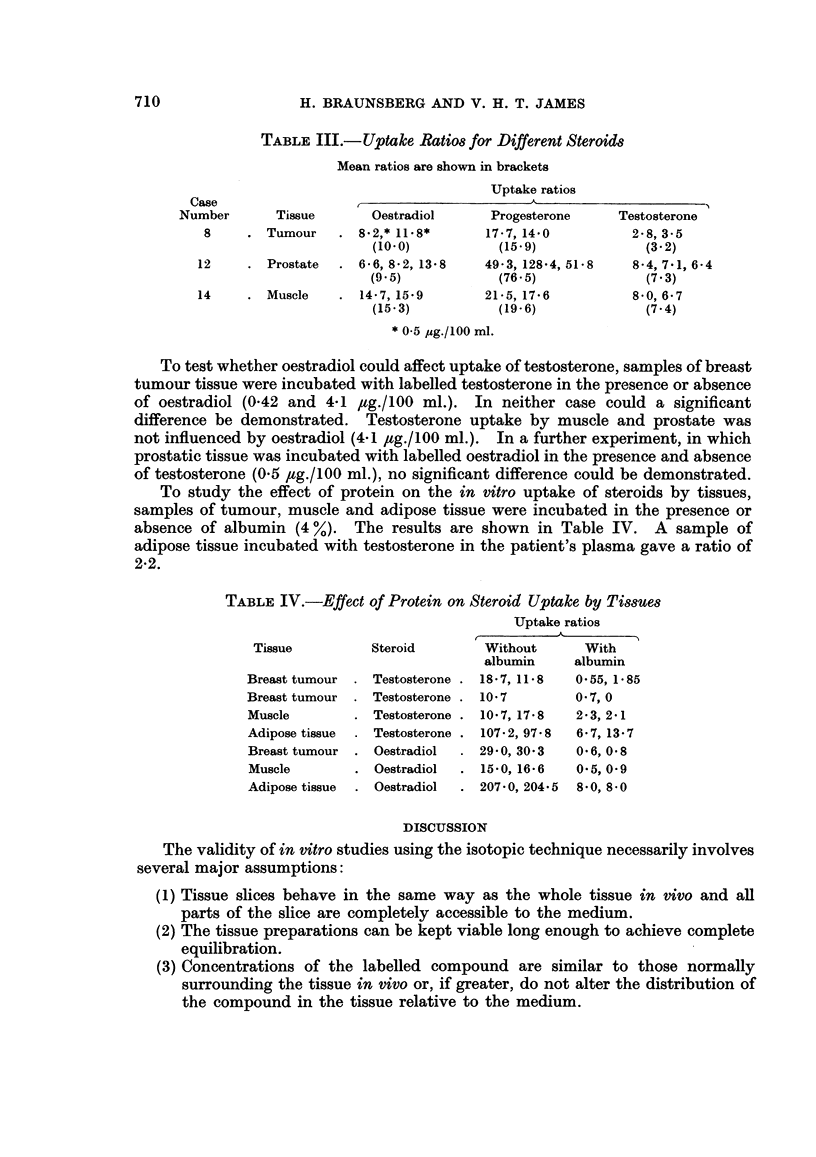

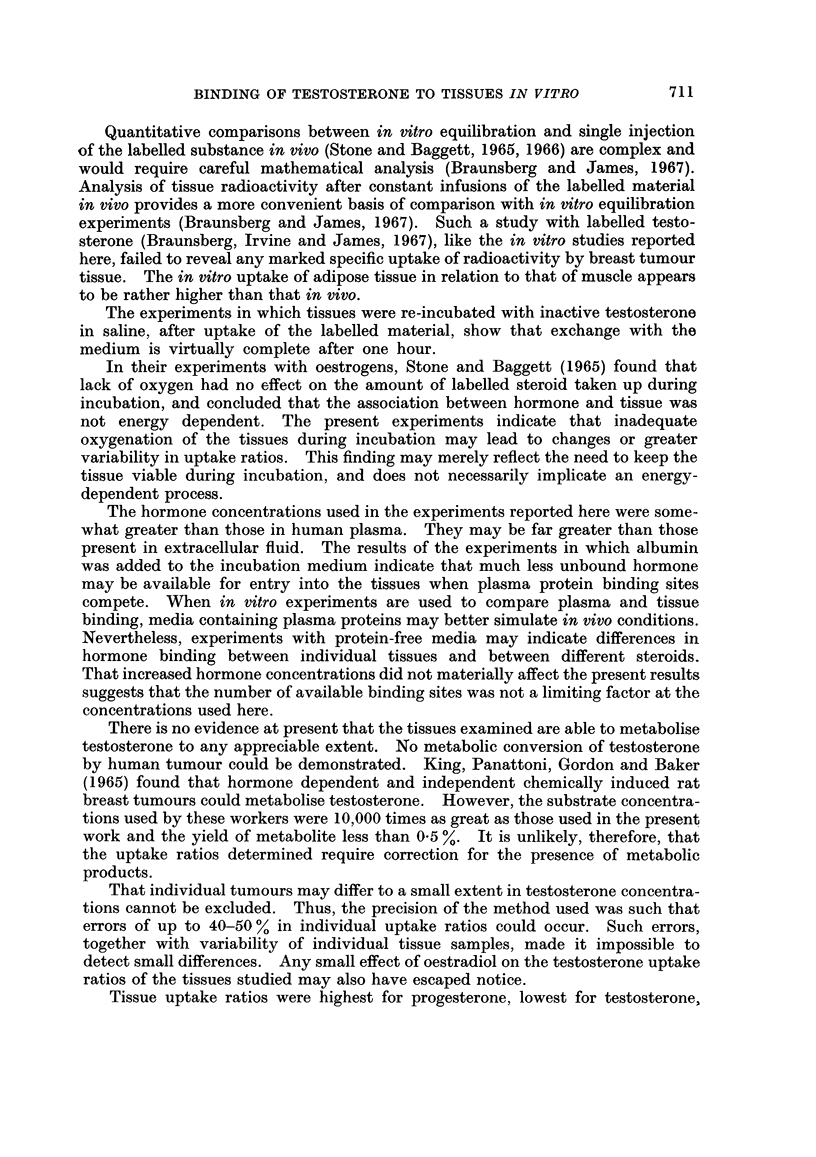

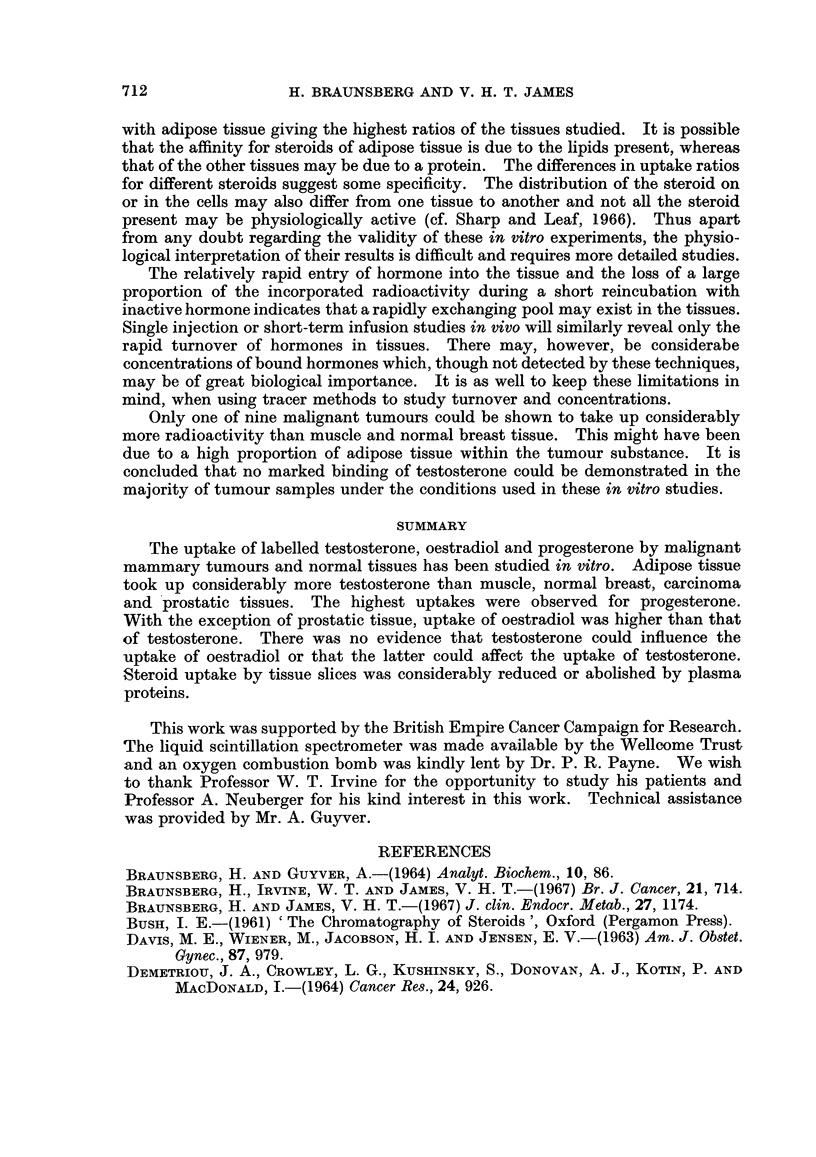

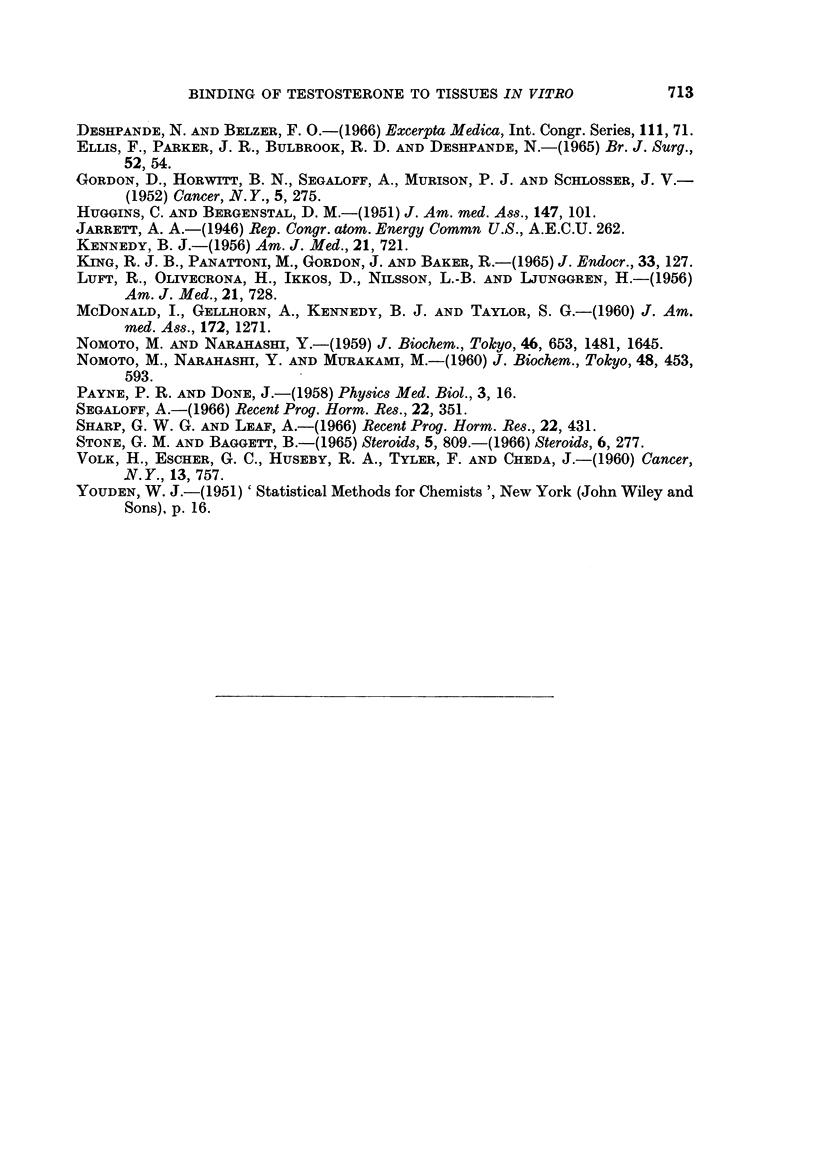


## References

[OCR_00755] Braunsberg H., James V. H. (1967). Mathematical analysis of experiments with labeled hormone tracers: problems of interpreting tissue radioactivity.. J Clin Endocrinol Metab.

[OCR_00759] DAVIS M. E., WIENER M., JACOBSON H. I., JENSEN E. V. (1963). ESTRADIOL METABOLISM IN PREGNANT AND NONPREGNANT WOMEN.. Am J Obstet Gynecol.

[OCR_00763] DEMETRIOU J. A., CROWLEY L. G., KUSHINSKY S., DONOVAN A. J., KOTIN P., MACDONALD I. (1964). RADIOACTIVE ESTROGENS IN TISSUES OF POSTMENOPAUSAL WOMEN WITH BREAST NEOPLASMS.. Cancer Res.

[OCR_00777] GORDON D., HORWITT B. N., SEGALOFF A., MURISON P. J., SCHLOSSER J. V. (1952). Hormonal therapy in cancer of the breast. III. Effect of progesterone on clinical course and hormonal excretion.. Cancer.

[OCR_00781] HUGGINS C., BERGENSTAL D. M. (1951). Surgery of the adrenals.. J Am Med Assoc.

[OCR_00788] IKKOS D., LJUNGGREN H., LUFT R., NILSSON L. B., OLIVECRONA H. (1956). Hypophysectomy in the treatment of malignant tumors.. Am J Med.

[OCR_00782] KENNEDY B. J. (1956). Extirpative endocrine therapy for advanced cancer of the breast.. Am J Med.

[OCR_00784] King J. B., Panattoni M., Gordon J., Baker R. (1965). The metabolism of steroids by tissue from normal and neoplastic rat breast.. J Endocrinol.

[OCR_00801] Segaloff A. (1966). Hormones and breast cancer.. Recent Prog Horm Res.

[OCR_00803] Sharp G. W., Leaf A. (1966). Studies on the mode of action of aldosterone.. Recent Prog Horm Res.

[OCR_00805] Stone G. M., Baggett B. (1965). The uptake of some tritiated estrogenic and non-estrogenic steroids by the mouse uterus and vagina in vivo and in vitro.. Steroids.

